# Features in Microfluidic Paper-Based Devices Made by Laser Cutting: How Small Can They Be?

**DOI:** 10.3390/mi9050220

**Published:** 2018-05-07

**Authors:** Md. Almostasim Mahmud, Eric J. M. Blondeel, Moufeed Kaddoura, Brendan D. MacDonald

**Affiliations:** 1 Faculty of Engineering and Applied Science, University of Ontario Institute of Technology, 2000 Simcoe Street North, Oshawa, ON L1H 7K4, Canada; md.mahmud@uoit.net; 2 ExVivo Labs Inc., 3 Regina Street North, Suite A, Waterloo, ON N2J 2Z7, Canada; eric@exvivo.ca (E.J.M.B.); moufeed@exvivo.ca (M.K.)

**Keywords:** paper-based devices, microfluidics, miniaturization, wicking, compact µPADs

## Abstract

In this paper, we determine the smallest feature size that enables fluid flow in microfluidic paper-based analytical devices (µPADs) fabricated by laser cutting. The smallest feature sizes fabricated from five commercially available paper types: Whatman filter paper grade 50 (FP-50), Whatman 3MM Chr chromatography paper (3MM Chr), Whatman 1 Chr chromatography paper (1 Chr), Whatman regenerated cellulose membrane 55 (RC-55) and Amershan Protran 0.45 nitrocellulose membrane (NC), were 139 ± 8 µm, 130 ± 11 µm, 103 ± 12 µm, 45 ± 6 µm, and 24 ± 3 µm, respectively, as determined experimentally by successful fluid flow. We found that the fiber width of the paper correlates with the smallest feature size that has the capacity for fluid flow. We also investigated the flow speed of Allura red dye solution through small-scale channels fabricated from different paper types. We found that the flow speed is significantly slower through microscale features and confirmed the similar trends that were reported previously for millimeter-scale channels, namely that wider channels enable quicker flow speed.

## 1. Introduction

Microfluidic paper-based analytical devices (µPADs) are an increasingly popular platform for medical diagnosis [1,2,3,4,5,6,7,8,9,10,11,12,13], environmental testing [14,15,16] such as monitoring the quality of water [17] and soil [18], detection of pathogens [19,20], metal-ion [21], pesticides in beverage and food [22,23,24], explosives [25], and other applications, such as the development of paper-based electronics [26,27]. One of the main advantages of µPADs is the capacity for low cost and portable diagnostic devices [28] for people who do not have access to traditional lab-based medical diagnosis, particularly in resource-poor regions, and for point-of-care devices. Paper is conducive to microfluidic devices because it is inexpensive, offers high surface area to volume ratio, provides a white background for colorimetric detection, can be disposed easily by burning, and enables wicking of fluid by capillary flow which eliminates the need for pumps. Paper types used in the fabrication of µPADs affect different design parameters of µPADs including: the size of the features in the device, the volume of reagents and sample absorbed by the device, sample flow behavior and spreading of reagents, chemical interference, required fabrication method, and time required to complete a test. In order for paper-based devices to provide widespread inexpensive access to diagnostic testing, it will be critical to have fabrication techniques capable of high precision mass production of small-scale features in µPADs.

The capability to generate small-scale features in µPADs reduces the cost for materials and reagents, and reduces the sample volume required to perform a test, enabling simpler sample collection methods such as finger prick [29] and microneedles [30,31,32,33,34,35]. Another advantage of compact devices is that multiple assays located on a single µPAD enables detection of many target analytes from a single sample. Previously, a laser cutting fabrication method with a foil backing was presented that generates narrow hydrophobic barrier widths of 39 ± 15 µm and microscale features with dimensions as low as 128 ± 30 µm in Whatman 1 Chr chromatography paper [36]. Other methods of fabrication are able to achieve varying resolutions for barriers and feature sizes in paper, for example on Whatman 1 Chr chromatography paper, channel widths of 184 ± 12 µm with barrier widths of 186 ± 13 µm were reported using the fast lithographic activation of sheets (FLASH) method [37], barrier widths of 850 ± 50 µm and channel widths of 561 ± 45 µm were reported with wax printing [38], channel widths of 500 ± 30 µm were reported using flexographic printing [39], and barrier widths of 380 ± 40 µm with channel widths of 670 ± 50 µm were reported using screen printing [40]. On nitrocellulose membrane a channel width of 100 µm with barrier widths of 86–118 µm were reported using wax-printing [41], drawn line-to-line channel widths of 150 µm with barrier widths of 85 ± 5 µm were reported using laser cutting and ablative etching [42], and an approximate channel width of 100 µm with a barrier width of 60 µm were reported using a laser-based direct-write technique [43]. The introduction of methods that can create increasingly smaller features in paper-based devices leads to the question: how small can the features be while maintaining the capacity for fluid flow? Paper is a fibrous matrix of cellulose that has a relatively random local composition except in cases where the paper is specially made in small batches at a higher cost [44]. Once the fabrication methods for paper-based devices reach a high enough level of precision, the smallest possible feature size in commercially available paper types will not be limited by the fabrication technique but will ultimately be limited by the physical structure of the fibrous matrix. The aim of this experimental work is to employ a fabrication technique with a high enough precision so the feature size is not limited by the fabrication, and determine the smallest feature widths that retain the capacity for fluid flow in five commercially available paper types.

Fluid flow in µPADs is capillary driven flow through the hydrophilic fibrous matrix [45,46] that is generally used to transport and mix a sample with reagents and for the reactions to yield a detectable signal readout. The capillary flow through the features of a µPAD influences the effective concentration of the analyte in the detection zones, the consistency of the analyte detection, and the time required to generate a result. Environmental conditions such as temperature and relative humidity can affect the fluid flow velocity through the paper channel [47,48] where higher temperature results in faster flow of fluid [47] and low relative humidity of the surrounding air can cause evaporation and eventual fluid dry out. Width has also been shown to influence fluid flow behavior, with wider channels resulting in faster fluid flow [47,48]. Past work has been conducted for flow through paper channels of millimeter scale widths [44,45,48,49,50,51,52,53] and centimeter scale widths [47]; however, as µPAD fabrication methods improve in resolution and precision, features can now be generated at the microscale. The characteristics of capillary driven flow through microscale features may be different due to the high surface area to volume ratios. Therefore, it is essential to know the behavior of flow through microscale channels to design compact µPADs that enable controllable and predictable flow of sample to the assays and uniform distribution of reagents throughout the devices.

In this experimental study, we determine the smallest feature sizes that will still enable fluid flow for five different types of paper: (i) Whatman 1 Chr chromatography paper (1 Chr), (ii) Whatman 3MM Chr chromatography paper (3MM Chr), (iii) Whatman regenerated cellulose membrane 55 (RC-55), (iv) Whatman filter paper grade 50 (FP-50), and (v) Amershan Protran 0.45 nitrocellulose membrane (NC). The features were generated using a previously reported foil-backed laser cutting technique [36] alongside a non-backed laser cut device for comparison. We correlate fiber width and the smallest feature sizes that enable fluid flow for the particular papers. We also report flow speed through small-scale channels fabricated from three different types of paper: (i) Whatman 1 Chr chromatography paper (1 Chr), (ii) Whatman 3MM Chr chromatography paper (3MM Chr), and (iii) Whatman regenerated cellulose membrane 55 (RC-55). This study answers the question of how small paper-based features can be while still enabling fluid flow, and reveals the fluid flow behavior for microscale features in µPADs.

## 2. Materials and Methods

### 2.1. Materials

Five different types of paper that are commonly used for µPADs, were selected for the experiments: Whatman 1 Chr chromatography paper (1 Chr), Whatman 3MM Chr chromatography paper (3MM Chr), Whatman regenerated cellulose membrane 55 of 0.45 µm pore size (RC-55) and Whatman filter paper grade 50 (FP-50) were purchased from VWR International (Mississauga, ON, Canada) and Amershan Protran 0.45 nitrocellulose membrane (NC) was purchased from Thermo Fisher Scientific (Mississauga, ON, Canada); all these types of paper are manufactured by GE healthcare. The thicknesses of 3MM Chr, 1 Chr, FP-50, RC-55 and NC paper type are 340 µm, 180 µm, 115 µm, 75 µm and 135 µm respectively. Allura Red AC (dye content 80%) was purchased from Sigma-Aldrich (Oakville, ON, Canada). Aluminum foil (Handi-foil Corp., Wheeling, IL, USA) and polystyrene petri dishes were purchased from UOIT central stores, Oshawa, Ontario. A roll of positionable mounting adhesive film 568 by 3M^TM^ (Maplewood, MN, USA) was purchased from Amazon.ca. All the dye solutions were prepared using distilled water and Allura Red dye.

### 2.2. Fabrication of Small-Scale Features

**Figure 1:**
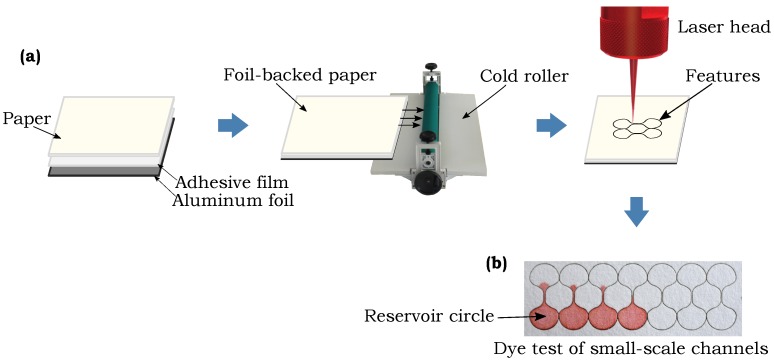
(**a**) Schematic of the fabrication technique used to generate small-scale features in the different paper types. (**b**) Overhead view of a dye flow test with the fabricated small-scale channels.

Microscale features were fabricated in the five different paper types using a previously reported method involving laser cutting of foil-backed sheets of paper [36]. Modifications to the previously presented method include use of a positionable mounting adhesive film (3M^TM^) in place of the double sided tape to affix the aluminum foil to the back of the paper and a manual cold roller machine (manual vinyl film mounting cold laminator, sold by ASC365 International Ltd., Toronto, ON, Canada, Amazon.ca) to bond the layers, as shown in Figure 1a. A 30 W CO_2_ laser (Speedy 100, Trotec, Wels, Austria) was used to create the barriers around the features through removal of the hydrophilic paper material. In previous use of this fabrication method [36] the resolution of the channels was reported as ±15 µm in 1 Chr, which is sufficiently smaller than the channel size so as not to influence the determination of the smallest feature size capable of fluid flow. After the modifications listed above, the channels fabricated for this work were found to have resolution slightly better than the previous work, which is sufficient so as not to influence the smallest feature size determination. We fabricated channels of different widths, ranging from 240 µm down to 120 µm line-to-line design width, which is the distance between the lines that are drawn in Inkscape and input into the laser to determine the path followed by the laser beam, with an interval of 20 µm. The actual widths of the channels that result on the paper material after cutting by the laser are smaller than the design widths due to the width of the laser beam cut, and we report and investigate these actual resulting widths in our analysis. To establish independence between the fabrication method and the smallest possible feature sizes in commercially available paper types, we also performed one set of experiments by fabricating the channels by laser cutting in 1 Chr paper without using the foil-backing technique. For this fabrication, the paper was cut without any adhesive or foil-backing and the channel was held in place by leaving connections to the main paper sheet, thus not fluidically isolating the channel from the rest of the paper sheet with the hydrophobic barrier, following a method similar to that described in a past work by Nie et al. [54].

### 2.3. Test Protocols

There were two different experimental objectives pursued in this study: (i) to determine the smallest features that enable fluid flow from the five different paper types, and (ii) to determine the flow speed of dye solution through the small-scale features.

In the experiments performed to address the first objective, all the channels of different widths (ranging from 240 µm to 120 µm of line-to-line design widths with an interval of 20 µm) were fabricated from each paper type in such a way that each channel connected two reservoir circles with a final shape that resembled a dumbbell, as shown in Figure 1b. The red dye solution (0.5 g/L Allura Red) was placed on the paper by bringing a saturated absorbent cotton rod in contact with a reservoir circle, as indicated in Figure 1b. A successful channel was deemed to be one where the dye solution wicked through to the opposite circle based on observation. The actual widths of the channel were measured with a microscope (OMAX 40X-1600X professional EPI-fluorescence trinocular biological microscope with 10MP USB digital Camera, sold by MicroscopeNet Canada, Kitchener, ON, Canada, Amazon.ca).

**Figure 2:**
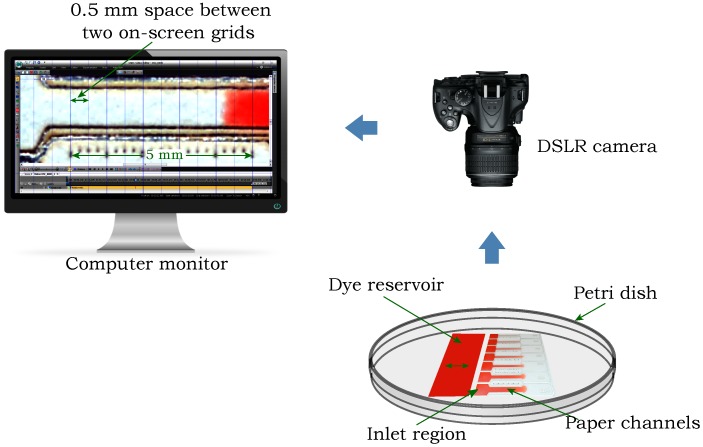
Schematic of the experimental procedure for measuring the flow speeds of dye solution through varying channel widths.

In the experiments performed to address the second objective, flow speeds of the dye solution were measured through small-scale channels of different widths fabricated from three of the different paper types: 1 Chr, 3MM Chr, and RC-55. A schematic of the experimental procedure is shown in Figure 2. The paper channels were located on a petri dish and fed from a rectangular dye reservoir made from 1 Chr that contained an excess volume of red dye solution (10 g/L, Allura Red) on it. The surface of the petri dish was hydrophobic which enabled the dye to move directly to the channel without spreading along the petri dish. The edge of the reservoir was brought in contact with the inlet regions of the channels and provided an abundant fluid supply for continuous flow through each channel. The petri dish was covered with its lid at the moment when the edge of the reservoir was brought in contact with the inlet regions of the channels to reduce the effect of the evaporation loss on the system. The flow was recorded with a DSLR camera (Nikon D5200 with Nikon Af-s Dx Micro 40 mm F2.8G lens, Nikon Corporation, Tokyo, Japan) which was connected with a PC to observe the flow on the monitor. A 5 mm scale with 250 µm tick marks was cut with the laser along each channel to measure the time required by the liquid front to travel a specific distance. To align the liquid front with the center of the cut tick marks, an on-screen grid software (MB-Ruler, version 5.3, Markus Bader—MB-Softwaresolutions, Iffezheim, Germany) was used that generates grids with a precise tick mark spacing, as shown on the computer screen in Figure 2. VSDC video editing software (Flash-Integro LLC., Vector Limited, Auckland, New Zealand) was used to measure the time required by the liquid front to travel between tick marks with millisecond increments.

## 3. Results and Discussion

### 3.1. Smallest Feature Size

**Figure 3:**
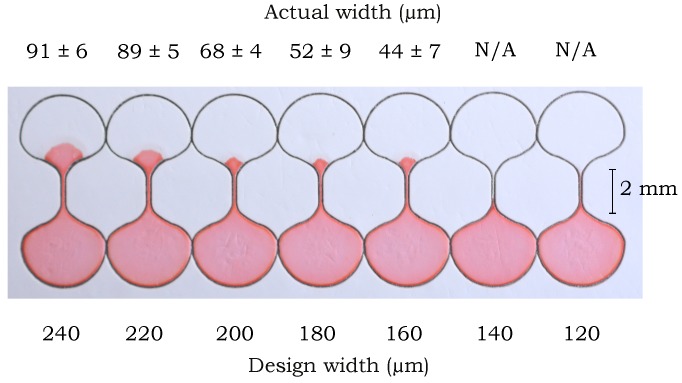
Testing small-scale channels of different width with dye solution to determine the smallest channel width that can be fabricated from a particular paper type. Channels fabricated from RC-55 are shown in the figure. The actual width is listed as N/A for cases where the channel failed to provide a continuous flow path and so no successful width could be listed.

To determine the smallest feature size in paper-based devices with the capability for fluid flow, we fabricated channels in the five different commercially available paper types listed above in Section 2.1 using the foil-backed technique, and fabricated channels in 1 Chr using the non-foil-backed method. We recorded the capability for fluid flow along a 1 mm long channel connecting two reservoirs, as shown in Figure 3. The tests were repeated in triplicate to establish repeatability and the results are summarized in Table 1. The smallest channel widths are the average of the smallest channel widths obtained from three repeated experiments for each paper type where the channels were measured at 20 points along their length. The ranges listed in Table 1 correspond to one standard deviation (±*σ*) of the 20 measurements from each of the three experiments (60 measurements total). From Table 1 we can see that NC offers the narrowest features, which is also the narrowest channel width achieved in NC to date, at 24 ± 3 µm, to the best of our knowledge [41,42,43]. Comparison of the foil-backed and non-foil-backed fabrication techniques show that the smallest width for successful fluid flow is similar, thus demonstrating independence from the specific fabrication method. It is still possible that different fabrication techniques may yield slightly different results for the smallest channel widths that enable flow. The widths in Table 1 answer the question of how small we can go, but we have not yet uncovered what is the cause of variation in the smallest feature sizes among the different commercially available paper types.

### 3.2. Influence of Fiber Width on Smallest Channel Widths

**Figure 4:**
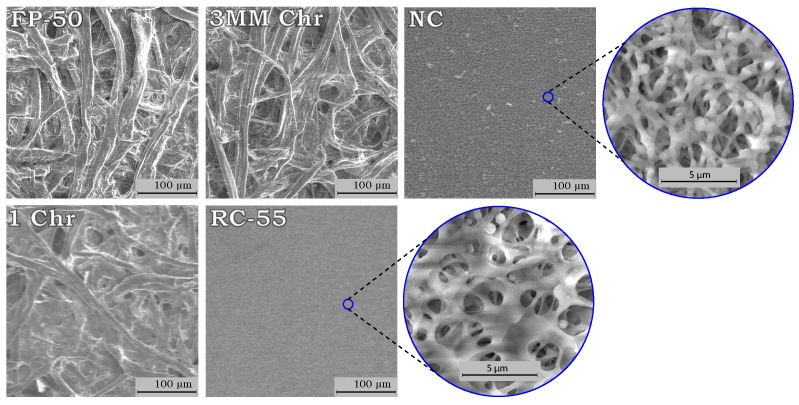
Scanning electron microscope (SEM) images of the fiber structure for the different paper types.

**Figure 5:**
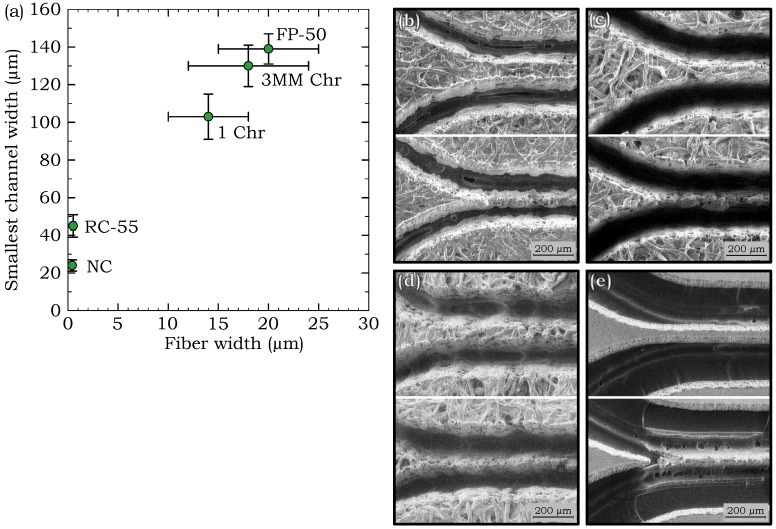
(**a**) The smallest channel widths in each of the paper types for successful fluid flow plotted against fiber widths. SEM images of the channels generated in the different paper types showing one intact channel with successful fluid flow (on the top) and one narrower channel where fluid flow was not successful (on the bottom), for (**b**) FP-50, (**c**) 3MM Chr, (**d**) 1 Chr, and (**e**) RC-55.

To understand which parameters influence the smallest features that enable flow in paper-based devices, we compared our data with some of the physical properties of the paper types and found that there was a correlation between the fiber width and the narrowest channel width for successful fluid flow. The average fiber width was determined from the widths of the fibers that can be observed in scanning electron microscope (SEM) images, as shown in Figure 4. Three SEM images taken at different locations were used to measure the fiber width of each paper type. A total of 150 fiber width measurements were taken from three SEM images, where the average of the measurements were taken as the fiber width and one standard deviation (±σ) of the measurements was used for the ranges, shown by the error bars in Figure 5a. The fiber widths of the five different paper types were plotted against the smallest channel widths in Figure 5a. The plot shows that a smaller fiber width correlates with a lower value of the smallest channel width. For successful fluid flow through a paper channel, the fiber structure should be continuously linked along the channel pathway to ensure that the fluid is wicked along by capillary forces. A channel generally fails to carry liquid when the fiber network along the channel becomes disconnected due to fibers that are loose or broken. The SEM images in Figure 5b–e show both successful and unsuccessful channels to illustrate how the fiber network becomes discontinuous as the channel widths are made too small. Therefore, the paper types with smaller fiber widths are capable of having continuous fiber networks along smaller channels and not disturbing the fluid pathway at smaller dimensions as compared to the larger fiber widths. Similar sized successful flow channels can be fabricated from both RC-55 and NC, with RC-55 shown in Figure 5e, because both of their fiber widths are less than 1 µm.

### 3.3. Flow Behavior through Microscale Channels

**Figure 6:**
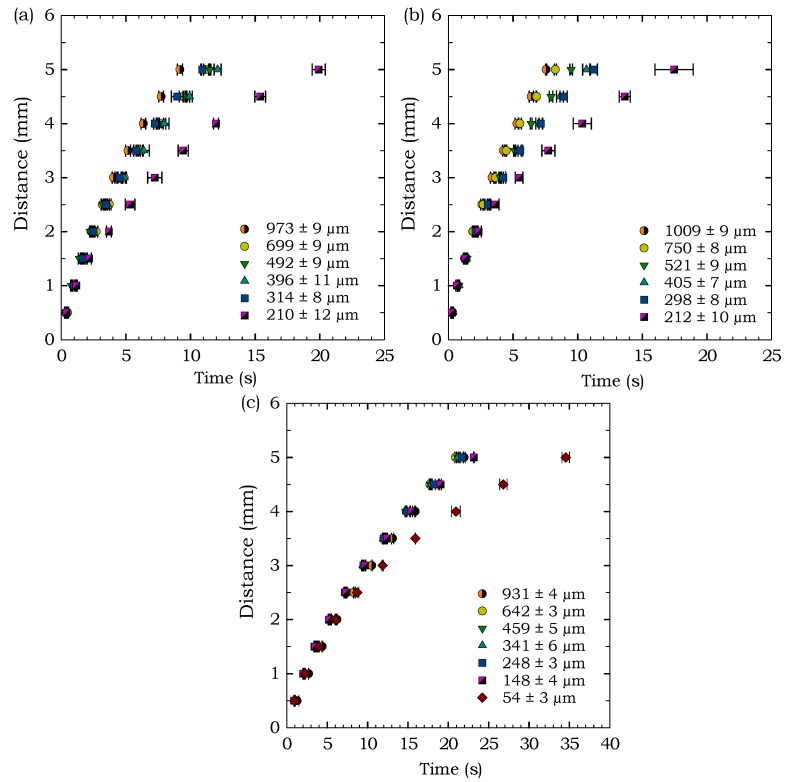
Flow behavior through varying channel widths fabricated in (**a**) 1 Chr, (**b**) 3MM Chr, and (**c**) RC-55.

To examine the flow behavior through microscale features in paper-based devices, we ran experiments using 1 Chr, 3MM Chr, and RC-55 and measured the time required for fluid to travel 5 mm with intervals of 0.5 mm, as shown in Figure 2. The results are shown only for these three paper types since the FP-50 is designed for flow-through filtration configurations and the NC exhibited unusual flow behavior, as described in Section 3.4 below, and thus the results for these two paper types were inconsistent. The line-to-line design widths and corresponding actual widths for the three paper types used in these experiments are summarized in Table 2. To measure the width of each channel, 20 measurements were taken along the length of the channel and the average value is reported in Table 2 with the range denoting one standard deviation (±σ). Figure 6 shows the travel time of the liquid front through various channel widths for 1 Chr, 3MM Chr and RC-55. The results in Figure 6 show a gradual increase in flow speed for increasing widths, except at the smallest widths, where the flow was substantially slower. These smallest width values are approximately the same size as the paper thickness in each of the three cases (thickness of 1 Chr: 180 µm, 3MM Chr: 340 µm, and RC-55: 75 µm). This indicates that the flow speeds do not vary as much when the channel width is substantially larger than paper thickness, but when the channel width and paper thickness are similar, the smaller width causes an appreciable reduction in the flow speed. Therefore, we observe a greater dependence of the channel width on the flow speed when the channel width is comparable to the paper thickness. Comparison with previous studies for significantly larger channels [44,47,48] also shows the same trend of quicker flows for wider channels in paper but with significantly quicker flow speeds compared to our microscale channels. Figure 6c shows that RC-55 also follows the same trend as the 1 Chr and 3MM Chr with the smallest channel experiencing slower flow speed; however, the overall flow speeds are slower than in the chromatography papers.

The smallest features were not included in the results in Figure 6 because the relatively long length of the channels (5 mm) was ∼50 times larger than the smallest feature sizes, which led to some inconsistent flow behavior for the smallest features. The 5 mm length was necessary to provide flow observations that could be compared to previous studies at substantially larger length scales [44,47,48], which used lengths of multiple centimetres with the lowest data points at 5 mm. The benefit of the smallest feature sizes is to create correspondingly small devices, so it is unlikely that the channels would flow a length of 50 times their width, and they were found to exhibit consistent flow speeds over shorter lengths (∼1 mm), as was observed during the smallest feature experiments described above in Section 3.1.

A frequently used equation to describe liquid flow through paper channel is the Washburn equation [55], where the porous medium is approximated as a bundle of parallel cylindrical capillaries. The Washburn equation indicates that the distance traveled by the liquid front in time *t* is expressed by:

(1) $$L\left(t\right)=\sqrt{\frac{rt\gamma cos\theta }{2\mu }}$$

where *L* is the distance travelled, *r* is the average pore radius, γ is the surface tension of the flowing liquid, θ is the contact angle between the capillary wall and the liquid, and μ is the dynamic viscosity of the liquid. The results in Figure 6 demonstrate that the flow behaves according to the $\sqrt{t}$ relationship as predicted by the Washburn equation. According to this equation, the distance travelled by the liquid front does not depend on the width of the paper channel, which was previously shown to be inaccurate by experimental studies [44,47,48], where flow through narrower channels was observed to experience a reduction in flow speed, and is further confirmed by our results for microscale features. Therefore, our work provides further confirmation that the Washburn equation alone is not sufficient for prediction of flows in paper-based microfluidic devices with features at smaller length scales, and highlights the importance of research exploring the complex dependence of the flow on various parameters, for various scenarios, such as the recent work by Castro et al. [48]. This study provides experimental data at the smaller length scales to help inform the future development of models to accomplish stronger predictive capacity.

### 3.4. Flow Behavior in Nitrocellulose Membranes

**Figure 7:**
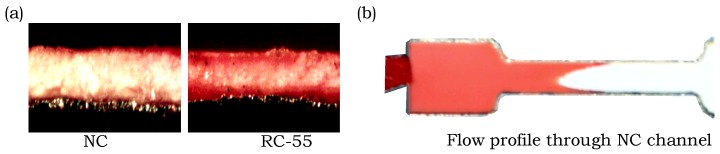
Photographs of the flow of red dye in NC and RC-55. (**a**) Cross-sectional views after red dye flowed through NC and RC-55. (**b**) Overhead view showing the concave flow profile of red dye in NC indicating faster flow along the sides of the channel.

Unusual flow behavior was observed in experiments with the Amershan Protran 0.45 nitrocellulose membrane (NC). NC and RC-55 have the same pore size of 0.45 µm, but in Figure 7a we can see that the red dye flows along the edges of the NC, whereas it is distributed more evenly through the entire thickness of RC-55. In Figure 7b we can see that flow along the edge of the NC channel is significantly faster than the middle area which generates a concave flow profile, whereas the chromatography paper, filter paper, and RC-55 have convex flow profiles. This behavior prevented us from making accurate measurements of the flow behavior in NC and may possibly be attributed to the fabrication method and chemical properties, since the laser may be burning or otherwise altering the hydrophilicity of the membrane along the sides of the channel.

## 4. Conclusions

We report the smallest channel widths that enable fluid flow in laser-cut paper-based devices for five different commercially available paper types. Specifically, the smallest widths were 139 ± 8 µm, 130 ± 11 µm, 103 ± 12 µm, 45 ± 6 µm, and 24 ± 3 µm fabricated from FP-50, 3MM Chr, 1 Chr, RC-55 and NC respectively. We also correlated the smallest channel widths with the widths of the fibers in different paper types and found that a smaller fiber width correlates with a lower value of the smallest channel width.

We investigated the flow speed of dye solution through the small-scale channels fabricated from different paper types. We found that the channels behave similarly to macro-scale channels with quicker flow speeds for wider channels but significantly slower overall speeds. The reported speeds will help in future modeling and design of µPADs with small-scale features.

Design of future µPADs will now benefit from the knowledge of the smallest features sizes reported herein, and situations where specific feature sizes are required can take advantage of the correlation between the fiber width and the smallest feature size. Specifically, in applications where smaller features are required, paper with smaller fiber widths can be used, such as NC or RC-55.

## Figures and Tables

**Table 1:** Smallest channel widths for successful fluid flow for five different commercially available paper types using the foil-backed (all paper types) and non-foil-backed (1 Chr only) technique.

Paper Type	Smallest Channel Width (µm)
FP-50	139 ± 8
3MM Chr	130 ± 11
1 Chr (w/o foil)	106 ± 11
1 Chr	103 ± 12
RC-55	45 ± 6
NC	24 ± 3

**Table 2:** Design widths and corresponding actual widths fabricated from different paper types used for flow speed measurements.

1 Chr		3MM Chr		RC-55	
Design Width (µm)	Actual Width (µm)	Design Width (µm)	Actual Width (µm)	Design Width (µm)	Actual Width (µm)
-	-	-	-	200	54 ± 3
300	210 ± 12	300	212 ± 10	300	148 ± 4
400	314 ± 8	400	298 ± 8	400	248 ± 3
500	396 ± 11	500	405 ± 7	500	341 ± 6
600	492 ± 9	600	521 ± 9	600	459 ± 5
800	699 ± 9	800	750 ± 8	800	642 ± 3
1100	973 ± 9	1100	1009 ± 9	1100	931 ± 4
